# A Rare Case of Childhood Glaucoma Resulting from Anterior Segment Dysgenesis Associated with a Homozygous Mutation in the *CPAMD8* Gene

**DOI:** 10.3390/genes17040494

**Published:** 2026-04-21

**Authors:** Nevyana Veleva-Krasteva, Kiril Genov, Kunka Kamenarova, Yoanna Kaneva, Kalina Mihova, Stanislava Kostova, Radka Kaneva, Alexander Oscar

**Affiliations:** 1 Department of Ophthalmology, Medical Faculty, Medical University of Sofia, 1431 Sofia, Bulgariaalekoscar@me.com (A.O.); 2 Eye Clinic, University Hospital “Alexandrovska”, 1431 Sofia, Bulgaria; kirilge@gmail.com; 3 Molecular Medicine Center, Department of Medical Chemistry and Biochemistry, Medical Faculty, Medical University of Sofia, 1431 Sofia, Bulgaria; kkamenarova@mmcbg.org (K.K.); kmihova@mmcbg.org (K.M.); kaneva@mmcbg.org (R.K.); 4 Laboratory of Genomic Diagnostics, Department of Medical Chemistry and Biochemistry, Medical Faculty, Medical University of Sofia, 1431 Sofia, Bulgaria

**Keywords:** childhood glaucoma, anterior segment dysgenesis, CPAMD8 gene

## Abstract

The term “childhood glaucoma” summarizes a heterogeneous group of diseases characterized by elevated intraocular pressure and associated optic nerve damage. Secondary glaucoma may develop based on non-acquired ocular anomalies, the most common of which are anterior segment dysgeneses. We present a rare case of infantile childhood glaucoma resulting from anterior segment dysgenesis due to a homozygous mutation c.1881delG, p.(Arg627Serfs*6), leading to loss of function in the *CPAMD8* gene.

## 1. Introduction

The term “childhood glaucoma” summarizes a heterogeneous group of diseases characterized by elevated intraocular pressure and associated optic nerve damage. Left untreated, glaucoma leads to severe impairment of visual function and accounts for approximately 5% of all cases of childhood blindness worldwide [1].

According to the widely accepted classification of the Childhood Glaucoma Research Network [2], childhood glaucoma is divided into: (1) primary glaucoma (primary congenital glaucoma and juvenile open-angle glaucoma); (2) secondary glaucoma (glaucoma associated with non-acquired ocular anomalies; glaucoma associated with non-acquired systemic diseases or syndromes; glaucoma associated with acquired conditions; and glaucoma following cataract surgery); and (3) glaucoma suspect.

The diagnosis of glaucoma in childhood requires the presence of two or more of the following criteria: intraocular pressure ≥ 21 mmHg; glaucomatous changes in the optic nerve such as increased cupping, focal notching, or a difference in cupping between the two eyes of 0.2 or more; corneal changes such as increased corneal diameter or Haab’s striae; and visual field defects consistent with glaucomatous optic nerve damage [2].

Large epidemiological studies on the overall incidence of childhood glaucoma in all its variants are lacking, mainly due to the wide range of diseases that may lead to its development. More information is available regarding the frequency of individual types of glaucoma, although the data are contradictory depending on the studied population (geographic region and ethnic group) and the criteria used for classification. Some authors report a predominance of primary forms, while others indicate that secondary glaucoma is more common [3,4,5]. This highlights the need for thorough knowledge of both primary and secondary childhood glaucoma, especially considering that the latter requires precise etiological diagnosis, greater diagnostic effort, and often a different clinical approach.

Secondary childhood glaucoma is associated with non-acquired ocular anomalies (Axenfeld–Rieger anomaly, Peters anomaly, aniridia, ectropion uveae, persistent fetal vasculature, oculodermal melanocytosis, posterior polymorphous dystrophy, microphthalmia, microcornea, and lens ectopia), non-acquired systemic diseases or syndromes (connective tissue syndromes such as Marfan and Stickler syndromes; metabolic diseases and phakomatoses), acquired conditions (uveitis, trauma, steroid treatment, tumors, and retinopathy of prematurity), or may occur after cataract surgery [2].

A special place among the causes of secondary glaucoma is occupied by anterior segment dysgeneses (ASD). These represent a clinically and genetically heterogeneous group of congenital ocular anomalies affecting the anterior segment structures—cornea, iris, ciliary body, anterior chamber angle, and lens—with more than 50% of affected individuals developing glaucoma [6,7]. Their clinical manifestations include megalocornea or microcornea, corneal opacities, iris hypotrophy or atrophy, polycoria, corectopia, posterior embryotoxon, iridocorneal strands, cataract, and lens ectopia.

Over the past decade, significant efforts have been made to uncover the molecular and genetic bases of ASD. Methods for early diagnosis and screening in at-risk populations and prevention of the irreversible glaucoma complications are being developed.

To date, 20 genes have been described whose mutations are associated with glaucoma linked to ASD. Interestingly, mutations in 12 of them are also responsible for the development of isolated childhood glaucoma (*PAX6*, *LTBP2*, *FOXC1*, *PITX2*, and *TEK/ANGPT1*) [7,8].

A common feature of many ASD diseases is disturbance of extracellular matrix (ECM) composition and abundance, although the specific underlying alterations remain mostly unidentified. ECM signaling has been postulated as being one relevant component of the networks involved in anterior segment development [9]. Most known ASD genes encode transcription factors, with some representing proteins associated with the ECM [8]. They are responsible for the differentiation of mesenchymal cells of neural crest origin, which are involved in the formation of distinct tissues and structures of the anterior segment of the eye. Extracellular vesicles, particularly exosomes, are key mediators of intercellular communication in the eye, facilitating the transfer of proteins, lipids, and nucleic acids between cells to regulate tissue homeostasis and remodeling [10]. The most common ASD genes associated with an autosomal dominant pattern of inheritance (the most common), are *PAX6*, *JAG1*, *PITX2*, *PITX3*, *FOXC1*, *FOXE3*, *BMP4*, and *COL4A1*. Another group of genes associated with an autosomal recessive inheritance includes *CYP1B1*, *LAMB2*, and *B3GALTL*. There is evidence that other genes are also responsible for ASD phenotypes.

ASDs are characterized by significant genotypic and phenotypic heterogeneity, resulting in variability of clinical findings even among members of the same family. There is a considerable overlap in the clinical presentation among different ASDs, the most common of which are Axenfeld-Rieger anomaly/syndrome, Peter’s anomaly/syndrome, aniridia, microcornea, and sclerocornea [2,7]. There are also variants that are referred to as unclassified anterior segment dysgenesis.

A relatively new gene whose mutations can lead to the development of both isolated dysgenesis and primary and secondary glaucoma associated with anterior segment dysgenesis is *CPAMD8*. Biallelic variants in *CPAMD8* (anterior segment dysgenesis 8, OMIM #617319) are associated with a highly heterogeneous phenotype and in some cohorts represent the second most common hereditary cause of childhood glaucoma after *CYP1B1* and juvenile open-angle glaucoma after *MYOC* [11]. Recently, a specific form of autosomal recessive ASD has been associated with variants in *CPAMD8* [12]. Unlike other ASD cases, none of the four reported cases with biallelic *CPAMD8* variants had glaucoma; only one case developed ocular hypertension at 49 years of age, presumably exacerbated by lens ectopia and retinal surgery. Subsequently, a case of congenital glaucoma associated with lens subluxation resulting from a homozygous frameshift variant in *CPAMD8* was reported [13]. Therefore, *CPAMD8* sequencing should be considered in the investigation of both pediatric and juvenile open-angle glaucoma, especially when associated with iris abnormalities, cataract, or retinal detachment.

We present a rare clinical case of a child with secondary glaucoma due to a homozygous c.1881delG, p.(Arg627Serfs*6) mutation in *CPAMD8*, a gene with still unclear function but recently associated with ASD, myopia, and lens ectopia, megalocornea in the absence of elevated intraocular pressure, and more recently with childhood and juvenile open-angle glaucoma [11,14]. Our case is interesting with the early onset of glaucoma, a rarely mentioned presentation in the medical literature.

## 2. Case Presentation

The patient is a 3-year-old boy. The child was born at full term, with birth weight of 3240 g, from an uneventful first pregnancy with vaginal delivery.

At 1 year of age, the child was referred to an ophthalmologist due to bilateral megalocornea. A subsequent examination under general anesthesia (GA) revealed bilateral changes in the anterior segment of the eye, including bilateral megalocornea, posterior embryotoxon, deep anterior chamber, partial iris hypoplasia, and iridodonesis. The measured intraocular pressure (IOP) was 23 mmHg for the right eye (RE) and 26 mmHg for the left eye (LE). No pathological fundus changes or glaucomatous optic nerve disk changes were detected. Due to the elevated IOP values in the presence of anterior segment abnormalities, treatment with a topical carbonic anhydrase inhibitor was initiated, resulting in good IOP control. At the follow-up examination one month later under GA, IOP values were 15 mmHg (right eye) and 16 mmHg (left eye), and antiglaucoma therapy was discontinued. Several regular outpatient ophthalmologic examinations followed, with recommendations for repeated examination under GA regardless of recorded IOP values below 21 mmHg. Due to intercurrent health issues, these examinations were postponed until 2 years and 7 months of age, when elevated IOP was detected again. At the same time, the parents reported frequent use of corticosteroids (topical and inhaled) due to upper respiratory tract infections in the last month. The last examination revealed:

VOD = 0.5 with −2.0/180 TOD = 22 mmHg (ICare tonometry)

VOS = 0.5 with −2.0/0 TOS = 22 mmHg (ICare tonometry)

**Figure 1:**
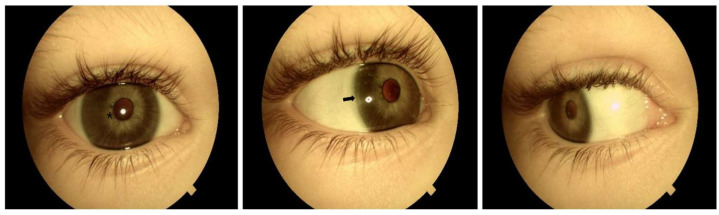
Right eye: Posterior embryotoxon is visible between the 6 and 12 o’clock positions (indicated by an arrow). The iris demonstrates diffuse hypoplasia (marked with asterisk), with a clearly visible pupillary sphincter and slight corectopia.

**Figure 2:**
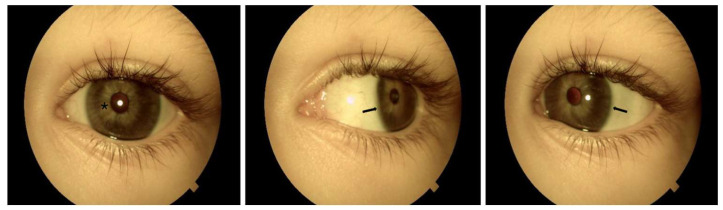
Left eye: Posterior embryotoxon is visible between the 1 and 11 o’clock positions (indicated by arrows). The iris demonstrates diffuse hypoplasia (marked with asterisk), with a visible pupillary sphincter and slight corectopia.

Both eyes show megalocornea without Haab’s striae, with corneal horizontal diameters of 14 mm for the right eye and 13.8 mm for the left eye, using a manual caliper under GA (the average horizontal corneal diameter in the general population is less than 12 mm). Posterior embryotoxon is visible from 6 to 12 o’clock in the right eye and from 1 to 11 o’clock in the left eye. No iridocorneal strands or anterior chamber angle pathology are observed. The iris shows diffuse hypoplasia with a visible pupillary sphincter and slight corectopia. Iridodonesis and mild ectopia of the transparent lenses were detected. All visible changes are present in Figure 1 and Figure 2. No pathological retinal changes or glaucomatous optic nerve head changes are detected (C/D 0.3). Axial length measures 21.70 mm (right eye) and 22.90 mm (left eye). Cycloplegic refraction reveals mixed astigmatism: OD +1.00/−3.0/31°; OS +2.25/−3.50/149°.

A fixed combination of a topical carbonic anhydrase inhibitor and beta-blocker was prescribed twice daily. One month later, a prostaglandin analog was additionally introduced.

Five months after initiation of antiglaucoma therapy, intraocular pressure is stable with the highest detected values of 15 mmHg.

The child was referred for genetic testing and consultation.

The study was conducted in accordance with the principles of the Declaration of Helsinki and was approved by the Ethics Committee of the Medical University–Sofia (Bulgaria). A written informed consent form was obtained from the patient’s parents.

**Figure 3:**
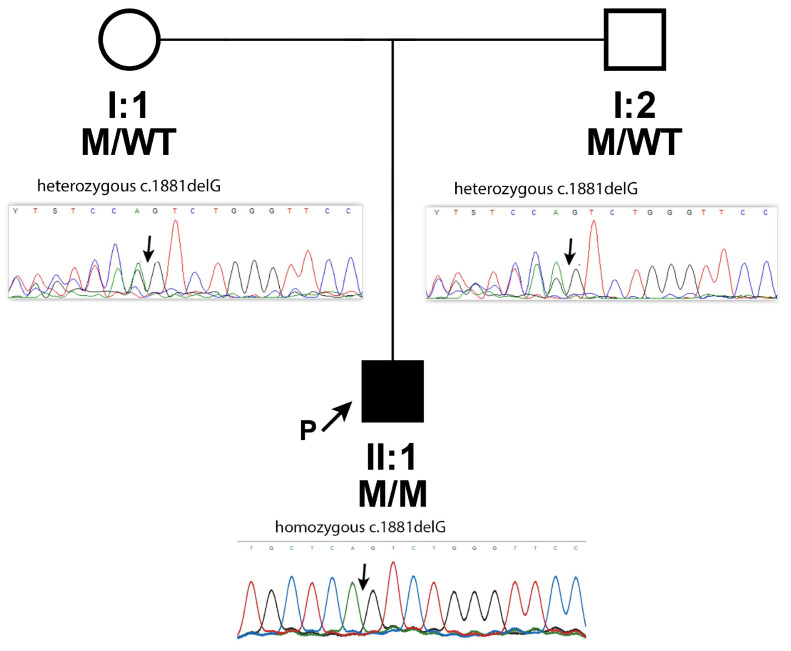
Pedigree drawing of family and sequencing chromatograms showing the segregation of a heterozygous deletion of one nucleotide G, c.1881delG, in exon 16 of *CPAMD8*, resulting in a loss-of-function allele p.Arg627Serfs*6 in patient II:1. Affected individuals are represented by filled symbols, while unaffected individuals are represented by unfilled symbols. Circles and squares denote females and males, respectively; P-proband. The proband is homozygous (bottom), and both parents show heterozygous genotypes (top); M: mutation c.1881delG.

Genomic DNA was extracted from leukocytes of the proband and his parents (Figure 3) using the Chemagic DNA blood 10 k kit H1 (Revvity chemagen Technologie GmbH, Baesweiler, Germany) and Chemagen Magnetic Separation Module (PerkinElmer^®^, Waltham, MA, USA) according to the manufacturer’s protocol. Whole exome sequencing (WES) of the index patient was performed according to the manufacturer’s protocol with a NovaSeq6000 instrument (Illumina, San Diego, CA, USA), and 2 × 150 bp paired-end reads.

For secondary analysis, identification of single-nucleotide variants from the human genome GRCh37/hg19 and quality filtering, we used a locally maintained DRAGEN server (Illumina). Variant calls were required to have at least 20× coverage (depth of 20 mapped reads) and a quality score of at least 20 (implying an accuracy >99%). For heterozygous genotypes, the alternative allele ratio (variant allele frequency, VAF) was set between 0.20 and 0.75 following variant quality control in similar studies and our in-house established protocol for WES analysis.

Variants were filtered according to the minor allele frequency (MAF) < 1% in the population databases Genome Aggregation Database (gnomAD), https://gnomad.broadinstitute.org/ (accessed on 17 April 2026), following variant quality control published in rare variant studies [15] and an internally established protocol for WES analysis. Variants with presumably disruptive impact on the protein, including splice acceptor variants, splice donor variants, stop gained, frameshift variants, stop lost, and start lost variants; missense variants (their pathogenicity depends on an amino acid change and a protein domain affected) and synonymous variants (they can also be functional because they can disrupt transcription, splicing, co-translational folding, mRNA stability, and can modulate gene expression by affecting transcription and splicing regulatory factors in protein-coding regions) were used for consideration.

The mutation disease database ClinVar (ncbi.nlm.nih.gov/clinvar/, accessed on 17 April 2026) was used to identify variants previously reported as pathogenic/likely pathogenic, and those described as likely benign/benign variants were discarded. The impact of missense variants was assessed using six predictor tools: SIFT, PolyPhen2, MutationTaster, MutationAssessor, FATHMM-XF, and FATHMM MKL Coding.

Finally, the potential pathogenicity of the selected variants was assessed according to the ACMG criteria [16].

After filtering, a homozygous variant in the *CPAMD8* gene (NM_015692.5, exon 16), c.1881delG (rs772486886), was identified in the patient.

This deletion creates a premature termination codon (p.Arg627Serfs*6) in the *CPAMD8* gene. It is expected to result in an absent or defective protein product. Loss-of-function variants in *CPAMD8* are known to be pathogenic [12]. The identified variant is rare, with an MAF of 0.00001928 (exomes) and 0.00001314 (genomes) (GnomAD), with no reported homozygotes. To our knowledge, *CPAMD8*-c.1881delG has not been reported in the literature in patients with *CPAMD8*-related diseases; in ClinVar, it has been classified as pathogenic (Variation ID: 1917220).

Furthermore, the DNA of the proband (II:1) and his parents (I:1 and I:2, Figure 3) was subjected to Sanger sequencing (3500XL Genetic Analyzer, Applied Biosystems, Foster City, CA, USA, Forward primer 5′ to 3′: tcccaaagtgttgggattaca, Reverse primer 5′ to 3′: ttggaatgagagatgggaaag) to (i) confirm the deletion c.1881delG identified by NGS in the proband and (ii) investigate its segregation in the parents. We found that the homozygous c.1881delG was inherited from his parents, who were both heterozygous, and passed it to the son (Figure 3).

The G deletion, resulting in a frameshift c.1881delG in exon 16, is classified as pathogenic based on ACMG criteria: very strong PVS1, assuming to disrupt gene function by leading to complete absence of the gene product by lack of transcription or nonsense-mediated decay of the altered transcript; moderate PM2, MAF is below the expected carrier frequency for recessive conditions in the general population (gnomAD, exomes, and genomes); PM3, detected in trans with a pathogenic variant (since parents are heterozygous for the variant); and supporting PP5, the patient’s phenotype is specific for the disease, assuming a single genetic etiology.

Copy number variations analysis did not identify genomic alterations that result in abnormal copies of one or more genes, which might be involved in the phenotype.

## 3. Discussion

The limited number of reported clinical cases, as well as the phenotypic heterogeneity characteristic of ASD, make it difficult to determine the typical clinical manifestations in carriers of mutations in the *CPAMD8* gene.

The literature predominantly describes phenotypes affecting mainly the iris and lens, with reported findings such as iris hypoplasia, iridodonesis, uveal ectropion, corectopia, iris transillumination, early cataract, and lens ectopia [11,12]. Rarer pathologies affecting the posterior segment of the eye in the form of retinal detachment have also been reported [11]. Data on refraction are limited, but there is a tendency towards high myopia, without excluding the presence of hyperopic refraction [11,12].

A characteristic of our patient is the presence of bilateral megalocornea, posterior embryotoxon, partial iris hypoplasia, iridodonesis with a slightly ectopic clear lens, and elevated intraocular pressure. Megalocornea and lens ectopia were also reported by Liu et al. [17]. They reported 8 Chinese patients, and in addition to the above-mentioned changes, they also described iris abnormalities such as iris atrophy and posterior iris bowing.

Interestingly, posterior embryotoxon (observed in our patient), which is characteristic of other anterior dysgeneses such as Axenfeld-Rieger anomaly/syndrome, has only been reported twice in carriers of *CPAMD8* mutations (to our knowledge). Once in the cohort of Siggs et al. [11] and a second time in the father of a patient with ASD [18]. The presence of posterior embryotoxon in the healthy population is well-known, but in addition to it, iridocorneal strands have been described in the father of the proband, raising the question of whether heterozygous carriers of *CPAMD8* variants may manifest with milder ocular changes [18,19].

Regarding the differential diagnosis with other syndromes affecting the anterior segment, such as Axenfeld–Rieger anomaly/syndrome, neither polycoria nor extraocular manifestations have been described in the literature in patients with *CPAMD8* mutations [11,12]. In contrast to *PAX6*-associated disorders, in which reduced vision is observed due to foveal hypoplasia and corneal epithelial stem cell deficiency, corneal opacity and foveal involvement are absent [12]. The mode of inheritance can also be added to the differences—autosomal recessive for *CPAMD8*, compared to the dominantly inherited *FOXC1*, *PITX2*, and *PAX6* mutations [2,7].

Undoubtedly, the main question that remains is the existence of a causal relationship between ASD and the development of glaucoma. Between 30 and 75% of patients with ASD develop glaucoma [7]. All patients with elevated IOP in childhood should be investigated for changes in the anterior segment of the eye. At the same time, all patients with ASD should be carefully monitored for high intraocular pressure (IOP). Clinical cases of patients with typical ASD findings but without elevated IOP and glaucoma have been described [12]. On the other hand, there are data on patients with various types of glaucoma in whom there are no anterior segment abnormalities. Cohorts of patients with *CPAMD8* mutations presenting with juvenile open-angle glaucoma (JOAG), primary open-angle glaucoma (POAG), primary angle-closure glaucoma (PACG), and pigment dispersion syndrome/pigmentary glaucoma without ASD have also been described [20,21].

Of course, cases of patients with glaucoma due to ASD are also presented, and undoubtedly, the largest study investigating the association of *CPAMD8* mutations and glaucoma is that of Siggs et al. [11]. The authors included 268 patients diagnosed with childhood or juvenile glaucoma and their relatives. Biallelic variants in *CPAMD8* were found in 5.7% of patients with congenital glaucoma and 2.1% of cases with juvenile glaucoma. The authors concluded that biallelic mutations in *CPAMD8* are the second most common cause of childhood glaucoma after mutations in *CYP1B1* and juvenile open-angle glaucoma associated with variants in *MYOC*.

Glaucoma penetrance among *CPAMD8* mutation carriers remains unclear. According to Siggs et al., it is 62.5% for the entire group and 43.8% at 10 years of age among individuals with *CPAMD8* [11]. These findings correlate with the data of Souzeau E et al. for patients with Axenfeld-Rieger syndrome, who found a penetrance for glaucoma in 58.5% of all patients, which was 42.9% at 10 years of age [22].

*CPAMD8* is expressed in the human eye from the 9th week of gestation onwards [12]. From the 9th to the 22nd week of gestation, *CPAMD8* expression increases in the lens and decreases in the retina, with strong expression observed in the iris and cornea at the 22nd week. In adults, the highest expression of *CPAMD8* is found in the corneal epithelium and ciliary body [17].

The pathways by which *CPAMD8* contributes to eye development remain unclear, but a recent publication by Escribano et al. in *Zebrafish* suggests an interaction between *cpamd8* and *adamtsl4*, suggesting a possible contribution of *CPAMD8* to the development of fibrillar structures in the anterior segment [23]. *CPAMD8* participates in ocular anterior segment development and in ECM organization, and its loss-of-function underlies a spectrum of recessive Glaucoma–ASD phenotypes associated with extracellular matrix disorganization ranging from isolated trabeculodysgenesis to varying degrees of iridocorneotrabeculodysgenesis with associated lens abnormalities in some cases [9].

The exonic variant c.1881delG identified in our patient is predicted to result in a loss-of-function allele through a frameshift, as skipping exon 16 would disrupt the reading frame. This mutation is located in the second α2-macroglobulin, A2M, domain (p.Arg627), and, if synthesized, would likely result in a truncated, nonfunctional protein, completely eliminating the O-methyl transferase, α-macroglobulin thioester, α-macroglobulin receptor, and Kazal-like domains. This would result in a nonfunctional protein and complete loss of expression of both protein copies.

Interestingly, the results of Li X. et al. show that biallelic truncation variants were more frequently associated with ASD and, accordingly, with a more severe and earlier onset phenotype, including iris and ciliary body hypoplasia and iridocorneal adhesion, while in POAG/PACG, biallelic missense variants were more common [20]. These findings were confirmed in other later studies [17,21]. This suggests that different variants/changes in the gene cause different functional impairments of the encoded protein. Knowing the relationship between variants/changes and their effect could be used to predict the presentation and severity of ocular phenotypes [20].

Early-onset glaucoma genes may show incomplete penetrance, and this might be a possible mechanism whereby these genes contribute to disease development. Although further studies are needed to understand how the mechanisms of pathogenesis contribute to ASD in our patient, the data presented above suggest that *CPAMD8* deficiency is the most likely genetic cause of the observed phenotype. It is also possible that environmental risk or other genetic factors have led to steroid-induced glaucoma.

The main limitation of this report is its single-case design, which limits the generalizability of the findings and does not allow definitive conclusions regarding the genotype-phenotype relationship in patients with *CPAMD8*-associated glaucoma.

Despite its limitations, this case highlights the clinical importance of recognizing the phenotypic features associated with variants in *CPAMD8*, as early identification may facilitate timely diagnosis, appropriate genetic testing, and improved management of patients with *CPAMD8*-associated glaucoma.

## 4. Conclusions

ASDs are characterized by great genetic and clinical heterogeneity. The risk of developing secondary glaucoma in early childhood is very high; therefore, all children with glaucoma should be screened for changes in the anterior segment of the eye, and all patients with ASD should have their intraocular pressure monitored regularly, regardless of their values at the first examination. *CPAMD8* mutations are associated with variable phenotypic expression; the changes may vary significantly and may not consistently correlate with glaucoma presence. Nevertheless, all patients, as well as all their healthy relatives carrying heterozygous mutations, should be closely monitored.

## Data Availability

The original contributions presented in this study are included in the article. Further inquiries can be directed to the corresponding author.
